# Targeting regulatory T cells by E7777 enhances CD8 T-cell–mediated anti-tumor activity and extends survival benefit of anti-PD-1 in solid tumor models

**DOI:** 10.3389/fimmu.2023.1268979

**Published:** 2023-10-27

**Authors:** Haider S. Mahdi, Mary Woodall-Jappe, Preeti Singh, Myron S. Czuczman

**Affiliations:** ^1^ Department of Obstetrics, Gynecology & Reproductive Sciences, Magee-Womens Hospital, University of Pittsburgh, Pittsburgh, PA, United States; ^2^ MWJ Scientific Consulting, Ipswich, MA, United States; ^3^ Clinical Development and Medical Affairs, Citius Pharmaceuticals, Inc., Cranford, NJ, United States

**Keywords:** Denileukin diftitox, cancer, immunotherapy, immune checkpoint, preclinical

## Abstract

**Introduction:**

Regulatory T cell (Treg)-targeting cancer immunotherapy aims to transiently deplete Treg cells in the tumor microenvironment, without affecting effector T cells (Teff), thus both enhancing anti-tumor activity and avoiding autoimmunity. This study evaluated whether adding E7777 (a new formulation of denileukin diftitox [DD]) improved the efficacy of anti-PD-1 antibody therapy. DD is a recombinant protein containing the hydrophobic and catalytic portions of diphtheria toxin fused to full-length human IL-2. E7777 has the same amino acid sequence and brief circulatory half-life as DD, but with greater purity and potency.

**Methods:**

Subcutaneous syngeneic murine solid tumor models (colon cancer CT-26 and liver cancer H22) were used to evaluate safety, efficacy, and overall survival with E7777 and anti-PD-1 antibodies, each administered as monotherapy or in concurrent or sequential combination. In Experiment 1, treatments were compared to assess anti-tumor activity at various time points, with tumors excised and dissociated and tumor leukocytes characterized. In Experiment 2, tumor growth, response, and overall survival were characterized for 100 days following a 3-week treatment.

**Results:**

E7777 administered in combination with anti-PD-1 led to significantly increased anti-tumor activity and durable, extended overall survival compared to either treatment alone. In both tumor models, the Treg cell infiltration induced by anti-PD-1 treatment was counterbalanced by co-treatment with E7777, suggesting potential synergistic activity. Combination therapy showed the most favorable results. Treatment with E7777 was safe and well-tolerated.

**Discussion:**

Combined E7777 and anti-PD-1 therapy was well tolerated and more effective than monotherapy with either drug.

## 1 Introduction

Immune checkpoint inhibitors such as anti-PD-1 have been shown to modulate immune-tumor cell interactions and suppress tumor proliferation (1). Because only a small fraction of patients show durable responses to checkpoint inhibitor monotherapy, research is ongoing to identify the best combination treatments to use with anti-PD-1 drugs (2, 3). Evidence indicates that the presence of regulatory T cells (Tregs) in the tumor microenvironment plays a role in patient resistance to anti-PD-1 immunotherapy (4). Therefore, one therapeutic approach is to eliminate Tregs in the tumor microenvironment to enhance anti-tumor response to anti-PD-1 therapy (5–10). Targeted Treg elimination can activate tumor-specific effector T (Teff) cells to improve immunotherapy efficacy (5). This approach has been evaluated with several Treg-modulating agents in combination with PD-1 inhibitors (11–17). However, it has been difficult to implement this treatment approach because long-term Treg suppression increases patient risk for autoimmunity and adverse events (7, 8, 10). For example, therapeutic monoclonal antibodies (6, 9, 10) generally possess prolonged half-lives, and those targeting Tregs are associated with autoimmunity and immunogenicity (18, 19). It is also essential that therapies not simultaneously deplete both Tregs and tumor-reactive Teff cells (10).

Decreased IL-2 availability reduces T cell proliferation and function. Denileukin diftitox (DD) is a recombinant protein that contains the hydrophobic and catalytic portions of diphtheria toxin (DT) fused to human IL-2 (20). DT-mediated cytotoxic activity predominantly affects cells that express the intermediate and high-affinity forms of the IL-2 receptor; the latter contains the inducible alpha subunit CD25. Compared to other T cell or natural killer cell types, only Tregs constitutively express the high-affinity IL-2 receptor, rendering them extremely sensitive to IL-2-based therapeutics (21). While CD25 is constitutively expressed on Tregs, other cells (activated T-lymphocytes and natural killer and B-cells) generally express it only in response to a stimulus (20–22). With a brief half-life (<2 hours), DD is selectively bound and internalized by CD25+ Tregs (23, 24).

Formerly known as ONTAK^®^, DD has biological activity against IL-2-expressing malignancies (20). It received accelerated and full United States Food and Drug Administration (FDA) approval for the treatment of cutaneous T cell lymphoma (CTCL) in 1999 and 2006, respectively (23). This included a requirement to improve manufacturing processes to increase product purity. When reformulated in 2009, this improved-purity substance was considered a new drug by the FDA, requiring a new Biologics License Application, and assigned a new development code (E7777; engineered IL-2-diphtheria toxin fusion protein DD).

E7777 maintains the same amino acid sequence as DD, but with improved purity and potency (24, 25). Considered against monoclonal antibodies (19), E7777 has a very brief half-life in circulation (<2 h in rodents and in humans), allowing for transient Treg depletion (23, 24, 26). Research shows that E7777 may selectively remove Tregs not only in circulation, but also from the tumor microenvironment, making it a potentially safe partner to anti-PD-1 inhibitors (24, 26). Phase 1 and 2 trials (2018, 2021) conducted in Japan in patients with relapsed/refractory CTCL and peripheral T-cell lymphoma (PTCL) showed that E7777 was efficacious and well-tolerated, regardless of tumor CD25+ expression status (24, 25). E7777 received subsequent Pharmaceuticals and Medical Devices Agency regulatory approval in Japan for CTCL and PTCL (27), and is currently under Phase 3 evaluation in the United States in patients with recurrent/persistent CTCL with CD25+ tumors (CD25+ ≥20%) (NCT01871727).

Early studies of the original DD formulation evaluated Treg depletion in patients with solid tumors, with positive results (28–34). In a phase 1 dose-escalation study (2005), DD was administered as a single intravenous (IV) infusion to 4 patients (ovarian [2], breast [1], and lung [1] cancers); treatment significantly reduced Tregs from 25% to 18% and increased IFN-γ+/CD8+ Teff cells from 21% to 37% (30). In a subsequent phase 1 study (2008), DD administered via intraperitoneal (IP) infusion to 9 patients with refractory ovarian cancer was well tolerated and reduced Tregs in the peripheral blood and ascites, suggestive of a decrease in immune suppression (33).

In a pilot study (2010), DD was given in sequential combination with high-dose IL-2 to patients with metastatic renal cell carcinoma (N=18, with DD administered either before or between IL-2 treatments). All regimens were safe and showed an overall tumor response of 33%, and a 34%-88% reduction in peripheral Tregs (28). Additional studies in acute myeloid leukemia, melanoma metastases, mycosis fungoides, and Sézary syndrome have shown similar results (29, 31, 32). Based on this support for the effectiveness of DD in targeting Tregs within peripheral blood and the tumor immune microenvironment, additional studies were warranted to explore the potential role of this molecule in combination with immune checkpoint inhibition, a therapy not yet available when earlier DD studies were conducted.

This preclinical study evaluated the *in vivo* therapeutic efficacy, tolerability, and overall survival of E7777 or anti-PD-1 monotherapy versus combination therapy with both drugs. Treatment was investigated in subcutaneous syngeneic murine solid tumor CT-26 colon cancer and H22 liver cancer models. CT-26 and H22 models are known to be sensitive and suitable for the evaluation of efficacy and immune pharmacodynamic changes following novel anti-cancer therapeutic regimens (35, 36). Outcomes were evaluated based on the treatment(s) used and administration schedules employed. Two sets of experiments were conducted: Experiment 1 evaluated anti-tumor activity at various timepoints following a 2- (CT-26) or 3-week (H22) treatment course, with tumors periodically dissociated and tumor leukocytes characterized by flow cytometry (FC) and immunohistochemistry (IHC). Experiment 2 evaluated the impact on tumor growth, anti-tumor response, and overall survival for 100 days following a 3-week treatment course. Both experiments included these 2 immunotherapies delivered individually and in combination.

## 2 Materials and methods

### 2.1 Mice

Female strain BALB/c mice (Mus musculus), aged 6-8 weeks at study initiation, were supplied by Shanghai Lingchang Biotechnology Co., Ltd. (Shanghai, China). For inclusion, all mice were healthy, genetically unmodified, and had not been subjected to any prior procedures. Mice were housed in irradiated polysulfone IVC cages with up to 5 mice per cage, maintained at 20-26°C, 40%-70% humidity, on a 12-hour light/12-hour dark cycle. Bedding was made from autoclaved crushed corncobs, changed weekly. Environmental enrichment strategies included cardboard cylinders, tissue paper, and polycarbonate tubes, and club houses.

All study testing was open (without blinding) and conducted at Crown Bioscience, Inc. (Taicaing Jiangsu Province, China). Experiment 1 was conducted between August 22, 2019 and September 26, 2019; Experiment 2 was conducted between May 21, 2020 and September 8, 2020. A protocol was prepared prior to conducting the studies but was not registered.

All procedures involving animal care and use were approved by Crown Bioscience and conducted in accordance with the regulations of the International Association for Assessment and Accreditation of Laboratory Animal Care. All data management and reporting procedures were in accordance with applicable Crown Bioscience Institutional Animal Care and Use Committee Guidelines.

### 2.2 Test and control treatments

For both experiments, E7777 was manufactured by BSP Pharmaceuticals S.p.A., Italy and supplied by Dr. Reddy’s Laboratories as a sterile, lyophilized powder (concentration, 300 μg/vial). The vehicle control consisted of saline solution. For Experiment 1, anti-mouse PD-1 (RMP1-14) was manufactured and supplied by BioXCell Therapeutics (New Haven, CT) as a 62.4-mg solution with a concentration of 7.18 mg/ml. For Experiment 2, anti-mouse PD-1 was manufactured and supplied by Crown Bioscience as a 37.5-mg solution with a concentration of 8.2 mg/ml. The latter formulation was used for Experiment 2 because internal studies documented an observed loss of quality/activity over time in the BioXCell anti-PD-1.

### 2.3 Cell culture and tumor inoculation

Treatments were tested in subcutaneous syngeneic murine solid tumor models (CT-26 colon cancer cells and H22 liver cancer cells). The CT-26 and H22 cell lines were authenticated using short tandem repeat and single nucleotide polymorphism assays. Tumor cells were maintained *in vitro* as monolayer cultures in RPMI-1640 medium, supplemented with 10% fetal bovine serum at 37°C, in a 5% carbon dioxide (CO_2_) atmosphere.

Prior to tumor cell inoculation, cells in exponential growth phase were harvested and quantified by cell counter. Treatments were administered in a laminar flow cabinet. Each mouse was inoculated subcutaneously in the right flank with either 5 x 10^5^ CT-26 tumor cells or 1 x 10^6^ H22 tumor cells, both in 0.1 ml of phosphate-buffered saline.

### 2.4 Experimental design

For both experiments, the day of randomization (and treatment initiation) was denoted Day 0; randomization used the matched distribution method (Study Director TM software, version 3.1.399.19). Randomization for Experiment 1 began when mean tumor size reached ~93 mm^3^ (CT-26) and ~94 mm^3^ (H22). For each tumor model, 144 mice were allocated into 6 groups of 24. No *a priori* sample size calculation was performed. No mice were excluded. The study was performed for 14 (CT-26) and 23 days (H22) post-randomization. Randomization for Experiment 2 began when mean tumor size reached ~95.8 mm^3^ (CT-26) and ~83.3 mm^3^ (H22). For each tumor model, 80 mice were allocated into 5 groups of 16. The study was performed for 100 days, including 22 dosing days (Days 0-22) and 78 days of dosing-free observation (Days 23-100).

### 2.5 Dosing regimens

For both experiments, E7777 was IV administered at 2.5 µg/mouse, and anti-PD-1 was IP administered at 100 µg/mouse; the vehicle (control) solution was IV-administered.

**Figure 1:**
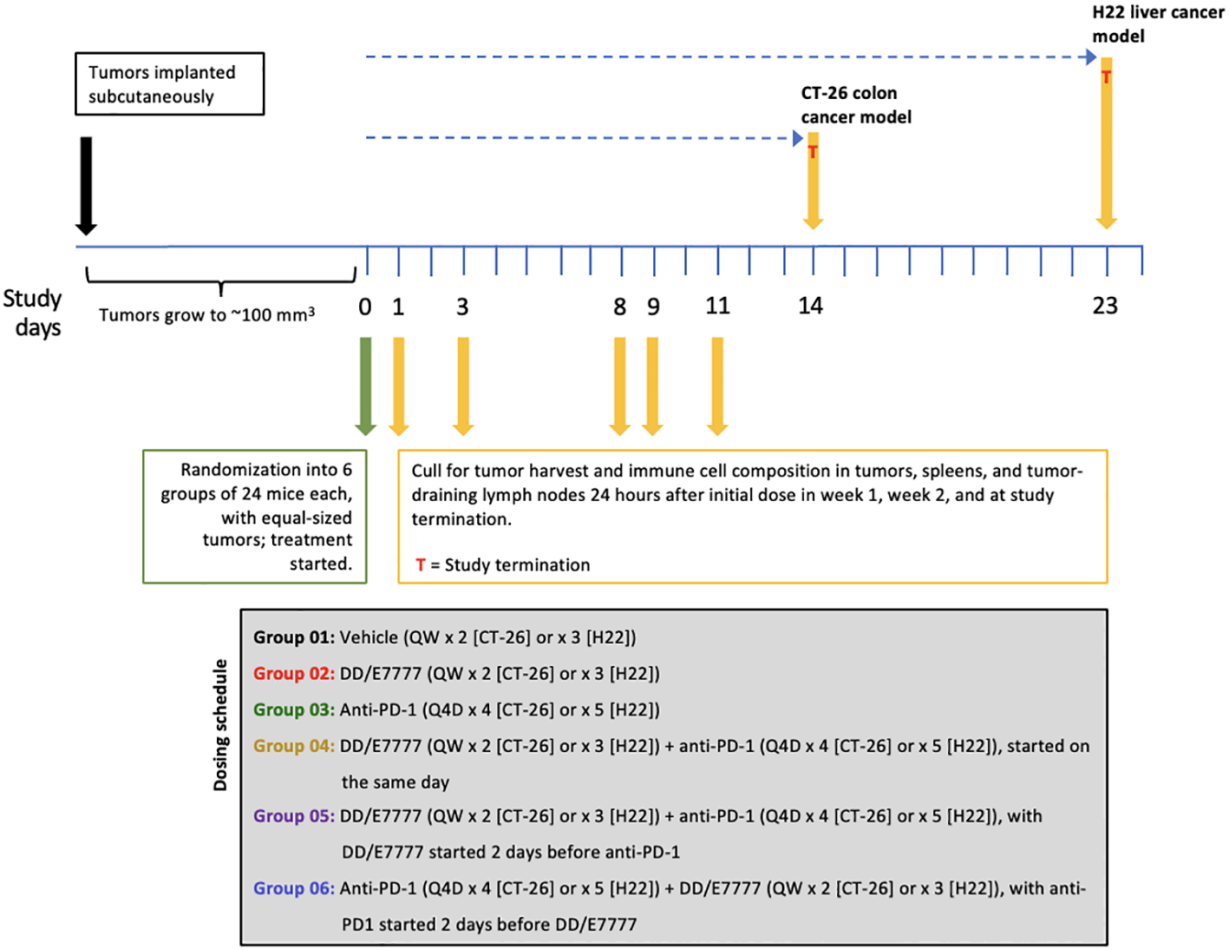
Outline of Experiment 1 study design and treatment schedule. Mice were dosed with E7777 or anti-PD-1 and collected for tumor harvest and immune cell composition^a^ according to the schedule shown below. ^a^ Because optimal administration schedules differed for the drugs, administration schedules were complex.

In Experiment 1, the dosing regimen for each group in both models was (**Figure 1**): Group 1. Vehicle (once weekly [QW] x 2 [CT-26] or x 3 [H22]); Group 2. E7777 (QW x 2 [CT-26] or x 3 [H22]); Group 3. Anti-PD-1 (every 4 days [Q4D] x 4 [CT-26] or x 5 [H22]); Group 4. E7777 (QW x 2 [CT-26] or x 3 [H22]) plus anti-PD-1 (Q4D x 4 [CT-26] or x 5 [H22]), started on the same day (concurrent administration); Group 5: E7777 (QW x 2 [CT-26] or x 3 [H22]) plus subsequent (sequential administration) dosing of anti-PD-1 (Q4D x 4 [CT-26] or x 5 [H22]) 2 days after; and, Group 6: Anti-PD-1 (Q4D x 4 [CT-26] or x 5 [H22]) plus sequential dosing of E7777 (QW x 2 [CT-26] or x 3 [H22]) 2 days after. In Experiment 2, the dosing regimen for each group in both models was as follows: Group 1. Vehicle (QW x 3); Group 2. E7777 (QW x 3); Group 3. Anti-PD-1 (Q4D x 6); Group 4. E7777 (QW x 3) plus anti-PD-1 (Q4D x 6), started on the same day; Group 5. E7777 (QW x 3) plus sequential dosing of anti-PD-1 (Q4D x 6) 2 days after.

Full treatment plans, including the allocated groups, assigned treatments, dose level, and dosing volume and frequency, are shown in **Supplementary Table 1** (**Supplemental Appendix**).

### 2.6 Observation and data collection

In both experiments, to reduce the potential for confounding after tumor inoculation, mice were checked daily for morbidity and mortality. Mice were also observed for any effects of tumor growth and treatment on behavior and appearance. This included mobility, food and water consumption, body weight gain/loss, and eye/hair matting. Body weight and tumor volume were measured in a laminar flow cabinet and recorded using Study Director™ software. Tumor volumes were measured in 2 dimensions using a caliper, with volume calculated as: V = (L x W x W)/2 (V=tumor volume; L=tumor length/longest tumor dimension; W=tumor width/longest tumor dimension perpendicular to L).

### 2.7 Study endpoints

Experiment 1 endpoints were: Percentage of tumor growth inhibition (TGI%) throughout the study, with TGI% expressed as 100 × (1-T/C), where T and C were the mean tumor volumes in the treatment and control groups, respectively, on a given day. Changes to TGI%, immune cell composition in tumors, spleens, and tumor-draining lymph nodes were measured within 24 hours of each group’s initial Week 1 dose (first data collection: 8 mice euthanized per group), Week 2 (second data collection: 8 mice euthanized per group), and at study end (Day 14 [CT-26] or Day 23 [H22]; third data collection and TGI% analysis: 8 mice euthanized per group).

To evaluate changes in multiple T cell subsets resulting from treatment, immune cell composition was characterized by FC and IHC. Resected tumors, spleens, and tumor-draining lymph nodes were dissected and dissociated into single-cell suspensions (for FC) or fixed, sectioned, and stained (for IHC). In FC, labeled antibodies were applied to the suspensions to identify cell subsets based on surface receptor expression: CD45+ hematopoietic cells were further characterized as CD3+ (pan-T); CD4+CD8- (T helper); CD4-CD8+ (Teff); CD4+FoxP3+ (Treg); CD4-CD8+ Tim3+LAG3+ T exhausted (Texh); and CD4-CD8+GzB+ T active effectors. IHC tissue sections were stained for visual evaluation of density and spatial relationships of Teff (CD8+) and Treg (FoxP3+) expression. Due to differing administration schedules, sampling times were chosen to address comparability, but were not identical across groups.

For IHC, all stained sections were scanned with the NanoZoomer-HT 2.0 Image system at 40x magnification (Hamamatsu Photonics, Hamamatsu City, Japan). All images were evaluated using the HALO^®^ image analysis platform (Indica Labs, Albuquerque, NM). In all cases, the full slide was evaluated, with large necrosis and stroma areas excluded. The analysis method used was dependent on staining pattern and target localization. If the staining pattern and target localization were clear, IHC-positive cells were counted and IHC scores were presented as the ratio of positive cell counts to total cell numbers. If the staining pattern was unclear, the IHC-positive expression area was measured, and IHC scores were presented as the percentage of the positive expression area.

Experiment 2 endpoints were TGI%, percentage of tumor response, number of mice with complete response, and survival time. The percentage of tumor response was expressed as 100 × (T/C). Complete response was defined as the tumor regressing to 0/undetectable at Day 100. Survival time was time to tumor volume reaching 3000 mm^3^.

In both experiments, mice were euthanized if they lost >20% of their weight relative to the first day of treatment, or had tumor volume >3000 mm^3^, or surface tumor ulceration ≥25%.

### 2.8 Statistical analysis

For both experiments, Bartlett’s test was used on prespecified days to compare tumor volumes and check the assumption of homogeneity of variance across evaluated groups. Unless otherwise specified, all tests were two-sided, with *P*-values <0.05 considered statistically significant.

If the *P*-value of Bartlett’s test was ≥0.05, a one-way analysis of variance (ANOVA) was conducted to test the overall equality of means across groups. If the ANOVA *P*-value was <0.05, additional *post-hoc* testing was performed (Tukey’s honest significant difference test for all pairwise comparisons, and Dunnett’s multiple comparison test to compare each treatment group with the vehicle group). If the *P*-value of Bartlett’s test was <0.05, Kruskal-Wallis one-way analysis of variance was used to evaluate the overall equality of medians among groups. If the *P*-value for the Kruskal-Wallis test was <0.05, treatment-versus-vehicle control comparisons were evaluated using Conover’s non-parametric test of variance with single-step *P*-value adjustment.

For Experiment 2, survival analysis was conducted using the Kaplan-Meier method, with survival defined as the time from randomization until death or the application of an ethical endpoint. Median survival time (MST) and increase in lifespan (ILS) were calculated for each study group. Kaplan-Meier curves were constructed for each study group, with log-rank testing used to compare groups.

All statistical analyses were conducted using R (version 3.3.1).

## 3 Results

### 3.1 Across all evaluated regimens, E7777 and anti-PD-1 were both well-tolerated by CT-26 and H22 tumor-bearing mice, with no observed mortality or serious adverse effects

Both CT-26 and H22 tumor-bearing mice treated with E7777 had a 10% to 15% mean body weight loss during the dosing period; this weight was recovered during post-dosing observation (**Supplementary Figure 1**). No other adverse events or deaths were observed.

### 3.2 Treg cell depletion with E7777 synergized with anti-PD1 therapy and prolonged survival in CT-26 and H22 models

**Figure 2:**
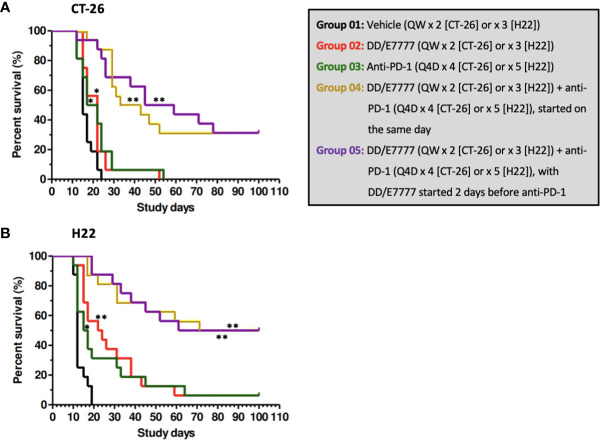
Kaplan-Meier survival curves of CT-26 model **(A)** and H22 model **(B)** treated with E7777 and anti-PD-1. Survival time was defined as time from randomization to death or tumor volume reaching 3000 mm^3^. Median survival time was calculated for each group, with log-rank testing to compare groups. For both cancer models, the differences in median survival time were significantly improved versus control for Groups 2, 3, 4, and 5. ^*^ *P* < 0.05, ^**^ *P ≤* 0.001.

**Figure 3:**
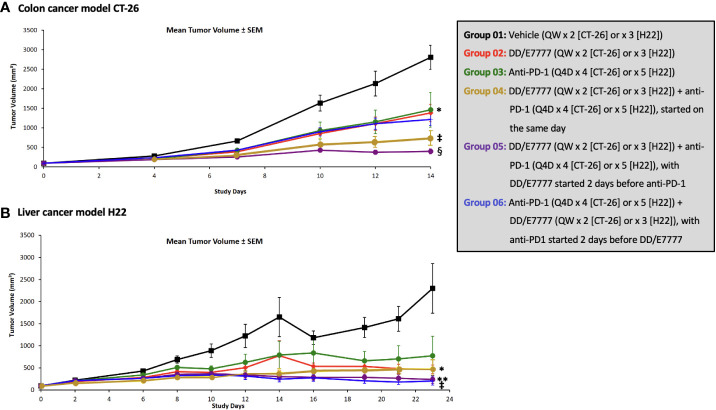
Effect of E7777 as single agent or combined with anti-PD-1 on mean tumor volume in subcutaneous syngeneic colon cancer model CT-26 **(A)** and liver cancer model H22 **(B)**. Tumor volumes were measured in 2 dimensions using a caliper, and Bartlett’s test was used on prespecified days to compare tumor volumes across evaluated groups. The differences in mean tumor volume were significantly improved versus control for the following groups at day 14 (CT-26) and day 23 (H22): For CT-26: Groups 3, 4, and 5; For H22: Groups 4, 5, and 6. ^*^ *P* < 0.05, ^**^ *P ≤* 0.01, ^‡^ *P ≤* 0.001, ^§^ *P ≤* 0.0001.

In both models, E7777 monotherapy had anti-tumor activity comparable to anti-PD1 monotherapy. E7777 combined with anti-PD1 significantly prolonged survival compared to monotherapy (**Figure 2**). When assessing the impact on tumor volume, sequential therapy (E7777 given first, followed by anti-PD1) showed more favorable anti-tumor activity in the CT-26 model compared to concurrent therapy or when anti-PD1 was administered first (**Figure 3**).

In Kaplan-Meier survival analysis (**Figure 2**, **Supplementary Table 2**) of CT-26 tumor-bearing mice, the MST of the vehicle control, single-agent E7777, single-agent anti-PD-1, E7777 plus anti-PD-1 co-administration, and E7777 plus anti-PD-1 sequential dosing, respectively, were 15 days, 22 days (*P*<0.05 vs vehicle control), 19.5 days (*P*<0.05 vs vehicle control), 38 days (*P*<10^7^ vs vehicle control), and 52 days (*P*<10^6^ vs vehicle control). In H22 tumor-bearing mice, the MST of the vehicle control, single-agent E7777, single-agent anti-PD-1, E7777 plus anti-PD-1 co-administration, and E7777 plus anti-PD-1 sequential dosing, respectively, were 12 days, 23 days (*P*<0.0001 vs vehicle control), 16 days (*P*<0.05 vs vehicle control), 85.5 days (*P*<10^7^ vs vehicle control), and 80.5 days (P<10^7^ vs vehicle control). For both tumor models, there was no significant survival difference between either treatment as monotherapy. In contrast, mice receiving combination treatments survived significantly longer than mice receiving either treatment alone (*P*<0.0001). There was no significant survival difference between mice in the combination groups.

### 3.3 In both the CT-26 and H22 models, combined E7777 and anti-PD-1 therapy showed improved anti-tumor activity

In the CT-26 colon model, both E7777 and anti-PD-1 therapy as single agents produced modest anti-tumor activity that did not achieve statistical significance compared to control. Anti-tumor efficacy was significantly enhanced in both the concurrent and sequential combination drug groups, and 31% of mice achieved complete and durable responses that lasted until study termination after 100 days. The mean tumor size of the vehicle control mice reached 2039.3 mm^3^ on Day 12 post randomization (**Supplementary Table 3**). Single-agent E7777 displayed modest anti-tumor efficacy against CT-26, with a TGI% of 20.9% on Day 12. No statistically significant difference (*P*>0.05) was observed compared to the control group. Single-agent anti-PD-1 also showed slight anti-tumor efficacy against CT-26, with a TGI% value of 9.6% on Day 12. Again, no statistically significant difference was observed compared to the control group. On the other hand, concurrent administration of E7777 and anti-PD-1 produced significant anti-tumor efficacy against CT-26, with a TGI% of 66.2% on Day 12 (*P*<0.001 vs vehicle control and individual monotherapies) (**Supplementary Table 3**). Sequential dosing of anti-PD-1 after E7777 produced significant anti-tumor efficacy against CT-26, with a TGI% of 53.9% on Day 12 (*P*<0.001 vs vehicle control and *P*<0.01 vs monotherapy groups). In the E7777 plus anti-PD-1 co-administration and sequential dosing groups, respectively, there were 5/16 and 5/16 complete responses.

In the H22 liver model, single-agent E7777 and anti-PD-1 produced statistically significant anti-tumor activity that was enhanced with sequential combination therapy, including 50% of mice achieving complete and durable responses that lasted until study termination after 100 days. The mean tumor size in the vehicle control mice reached 3029.4 mm^3^ on Day 12 post-randomization (**Supplementary Table 3**). Single-agent E7777 produced significant anti-tumor efficacy against H22, with a TGI% of 45.9% on Day 12 (*P*<0.01 vs vehicle control). Single-agent anti-PD-1 also showed anti-tumor efficacy, with a TGI% of 38.1% on Day 12 (*P*<0.01 vs vehicle control). Concurrent E7777 and anti-PD-1 administration produced significant anti-tumor efficacy against H22, with a TGI% of 59.8% on Day 12 (*P*<0.001 vs vehicle control) (**Supplementary Table 3**). However, no statistically significant difference was observed between this regimen and the corresponding monotherapy groups. In contrast, sequential administration of anti-PD-1 after E7777 produced significantly stronger anti-tumor efficacy against H22 compared to either monotherapy, with a TGI% of 70.4% on Day 12 (*P*<0.001 vs vehicle control and *P*<0.01 vs each monotherapy group). Complete response was identical in the co-administration and sequential groups (8/16 and 8/16).

### 3.4 E7777 combined with anti-PD-1 induced CD8 T-cell infiltration and reduced anti-PD-1-mediated Treg induction in the tumor immune microenvironment

**Figure 4:**
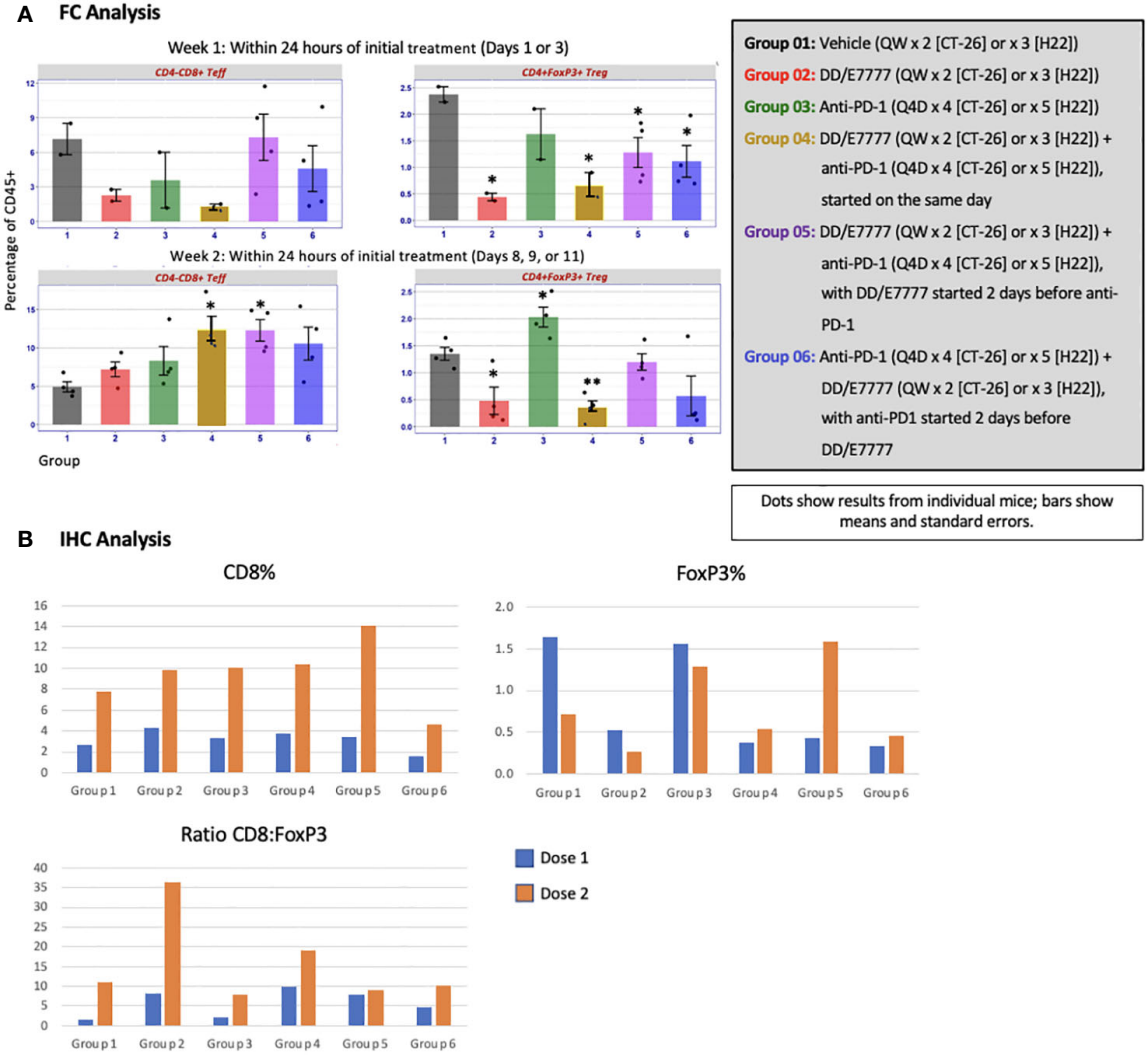
Evaluation of CD4-CD8+ Teff and CD4+FoxP3+ Tregs in tumors 24 hours after first and second E7777 treatment in colon cancer model CT-26 according to **(A)** FC analysis and **(B)** IHC analysis. Immune cell composition was characterized by FC and IHC to evaluate changes in multiple T cell subsets resulting from treatment. Anti-PD-1 treatment increased CD8+ Teff infiltration into tumors, but also induced increased Tregs, which the addition of E7777, on any schedule, helped to inhibit. In FC analysis, after the first E7777 treatment, there were no significant differences in CD4-CD8+ Teffs versus control, while CD4+FoxP3+ Tregs were significantly reduced versus control in Groups 2, 4, 5, and 6. After the second E7777 treatment, CD4-CD8+ Teffs were significantly increased versus control in Groups 4 and 5, while CD4+FoxP3+ Tregs were significantly reduced versus control in Groups 2 and 4. In contrast, CD4+FoxP3+ Tregs were significantly increased versus control in Group 3 after the second E7777 treatment. ^*^ *P* < 0.05, ^**^ *P ≤* 0.001.

**Figure 5:**
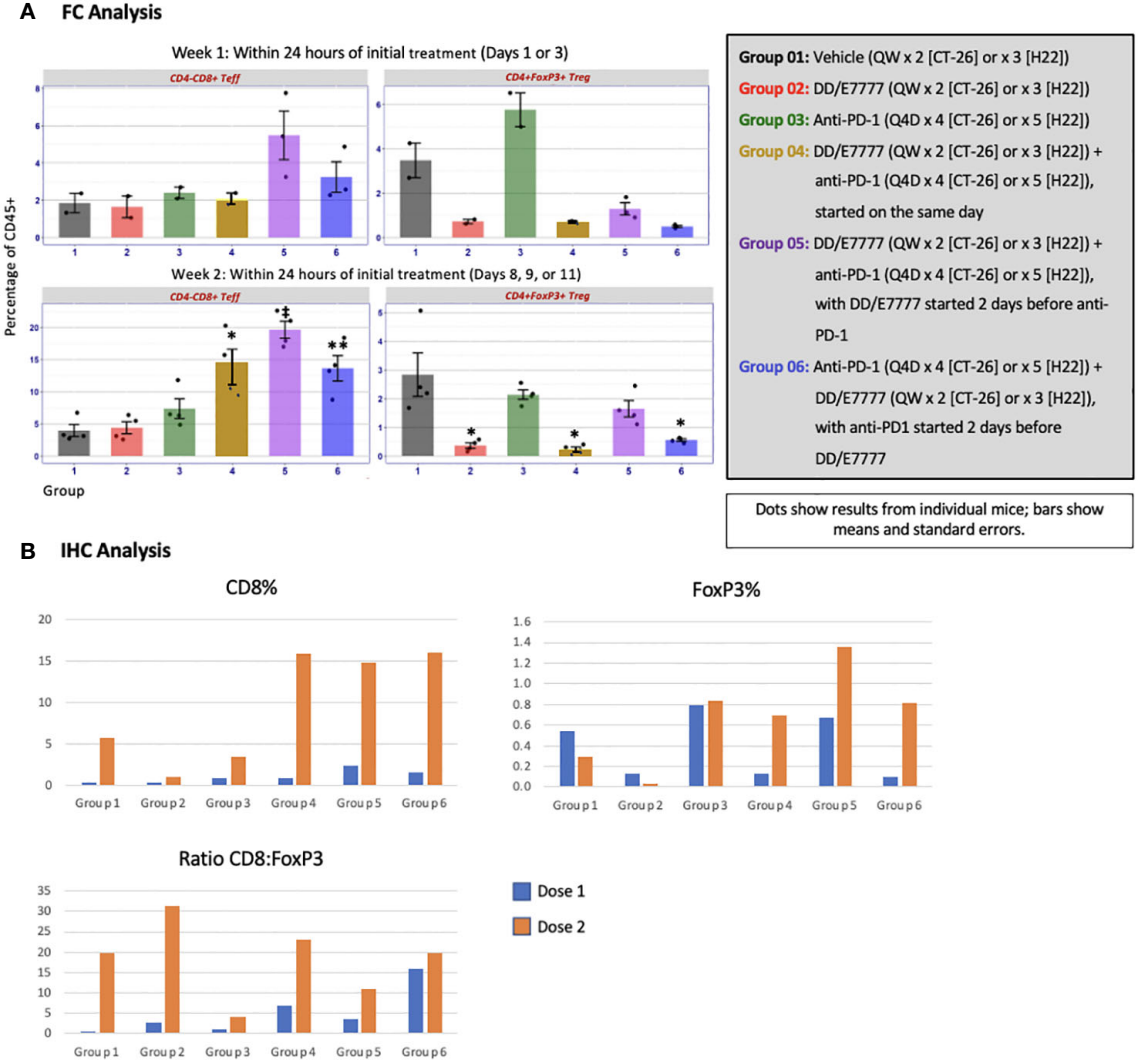
Evaluation of CD4-CD8+ Teff and CD4+FoxP3+ Tregs in tumors 24 hours after first and second E7777 treatment in liver model H22 according to **(A)** FC analysis and **(B)** IHC analysis. Immune cell composition was characterized by FC and IHC to evaluate changes in multiple T cell subsets resulting from treatment. CD8 T-cells in all combination groups increased by more than 2-fold compared to controls by week 2, whereas Tregs decreased by ~2 fold. Results characterized by IHC were consistent with FC data. In FC analysis, after the first E7777 treatment, there were no significant differences in CD4-CD8+ Teffs versus control or CD4+FoxP3+ Tregs versus control. After the second E7777 treatment, CD4-CD8+ Teffs were significantly increased versus control in Groups 4, 5, and 6, while CD4+FoxP3+ Tregs were significantly reduced versus control in Groups 2, 4, and 6. ^*^ *P* < 0.05; ^**^ *P ≤* 0.01; ^‡^ *P ≤* 0.0001.

The impact of E7777 and anti-PD-1 treatment on CD8 T-cells and Tregs was assessed during weeks 1 and 2 of therapy using both FC and IHC (**Figure 4** [CT-26]) and **Figure 5** [H22]). In both tumor models, anti-PD-1 treatment increased CD8+ Teff infiltration into tumors, but also induced increased Tregs, which the addition of E7777, on any schedule, helped to inhibit. In FC evaluation of combined E7777 and anti-PD-1 therapy (**Figures 4A**, **5A**), CD8 T-cells in all combination groups increased by ≥2-fold compared to control by week 2, whereas Tregs decreased by ~2 fold. However, at study termination, CD4+FoxP3+ rebounded in tumors in response to immune stimulation (**Supplementary Figure 2** [H22 model]). Results characterized by IHC (**Figures 4B**, **5B**) were consistent with FC data (see **Supplementary Figures 3, 4** for IHC staining images and **Supplementary Figure 5** for representative fluorescence activated cell sorting [FACS] plots). Tumors from mice who received combination treatment generally displayed a higher proportion of CD8+ cells than mice treated only with anti-PD-1. Mice treated with anti-PD-1 monotherapy also showed substantially elevated Tregs in the tumor immune microenvironment.

### 3.5 Immune cell ratios favoring Teff in tumors were generally improved using concurrent or sequential combination therapy

**Figure 6:**
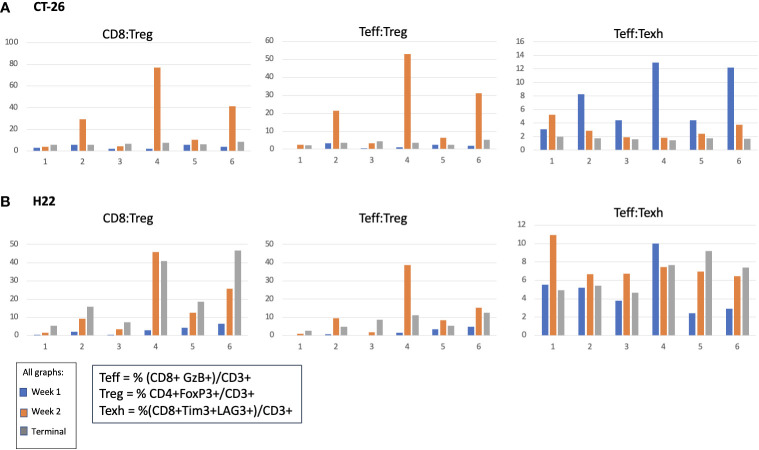
Effect of E7777 on tumor biomarkers following doses 1 and 2 characterized by FC for **(A)** colon cancer model CT-26 and **(B)** liver cancer model H22. Immune cell composition was characterized by FC to evaluate changes in multiple T cell subsets resulting from treatment. In the CT-26 model, E7777 improved CD8:Treg and related Teff:Treg tumor ratios, especially after the second dose. For the H22 model, CD8:Treg levels increased in all groups over the course of the study.

In the CT-26 model, E7777 improved CD8:Treg and related Teff : Treg tumor ratios, especially after the second dose (**Figure 6A**). Treatments featuring E7777 consistently showed the strongest effects on CD8:Treg, Teff : Treg, and Teff : Texh ratios. For the H22 model, increased CD8:Treg levels increased in all groups over the course of the study. (**Figure 6B**).

## 4 Discussion

The primary goal of Treg-targeted cancer immunotherapy is to transiently deplete Treg cells within the tumor microenvironment, without affecting Teff cells, while avoiding autoimmunity (10). In this murine analysis of syngeneic liver and colon solid tumor models, E7777 administered in combination with anti-PD-1 inhibitors led to significantly increased anti-tumor activity (as assessed by both FC and IHC) and durable, extended overall survival compared to either treatment as monotherapy. In both models (H22 liver and CT-26 colon), anti-PD-1 treatment alone led to increased tumor-associated Tregs, while adding E7777 transiently blocked this anti-PD-1-induced Treg increase. E7777 was also safe and well-tolerated.

While anti-PD-1 treatment was observed to induce an increase in tumor-associated Tregs, the role of PD-1/PD-L1 in Treg signaling is not fully understood (12). Murine and human studies indicate that PD-1 expression is associated with Treg dysfunction (12, 37, 38), and that PD-1 blockade may promote Treg function and proliferation (39), as observed in the current analysis. This was also shown in a study of patients with gastric cancer treated with anti-PD-1 therapy wherein hyperprogressive disease was associated with higher counts of proliferative effector CD45RA-CD25^hi^FoxP3^hi^ Tregs in the tumor microenvironment. Concurrent *in vitro* analysis of tissue samples confirmed that Treg proliferation occurred with anti-PD1 blockade (39). In the current study, adding sequential or concurrent E7777 transiently reduced anti-PD1-mediated Treg induction in both tumor models.

In the CT-26 model, E7777 treatment led to increased CD8+ cells in tumors and improved CD8:Treg ratios at both sample times. In the H22 liver model, tumor assessment after combination therapy showed significant tumor growth inhibition and greater Treg reductions at study termination versus monotherapy or control, substantial increases in CD8+ cells after the second dose, and a rebound in FoxP3+ levels by the third dose in response to immune stimulation. Further analysis of T-cell subsets provided additional mechanistic insights, with observed changes to immune cell ratios that included increased tumor CD8:Treg levels, identifiable after one dose, and increased tumor T active eff:Treg and Teff : Texh levels, noted after the second dose. In both study models, stronger beneficial effects were observed after 2 weeks of treatment compared to 1 week. It will be important to explore more sustained administration schedules and potential impacts on T-cell subsets in future research.

The current study findings align with prior, similar research (2018) that used a mouse melanoma model to evaluate an IL-2 receptor-linked diphtheria fusion toxin (with a slightly modified amino acid sequence) administered prior to anti-PD-1 antibodies. Compared to monotherapy, sequential treatment with either fusion toxin followed by anti-PD-1 led to reduced tumor growth, measured at several timepoints in the ~25 days following tumor injection (40).

This analysis also found only moderate adverse effects and no treatment-related deaths with E7777 administration, similar to prior findings. In phase 1 and 2 studies conducted in Japan (2018, 2021), the most commonly observed adverse events were elevated aspartate and aminotransferase, hypoalbuminemia, and lymphopenia in the phase 1 study (24), and neutropenia, thrombocytopenia, leukocytosis, and anemia in the phase 2 study (25).

Certain limitations exist in terms of interpreting and predicting how mouse immune response translates to human immunity. Syngeneic mouse models have evolved, but still do not represent the heterogeneity in the human cancer microenvironment. For example, injecting a bolus of cultured cancer cells subcutaneously does not capture the complexities of spontaneous tumor development in deeper tissues. Human proof-of-concept studies are currently underway to gain further understanding of these processes. An additional limitation of this study is the lack of functional assessment of Tregs and CD8 T-cells.

Although anti-PD-1 antibody treatment represents one of most important advances in solid tumor management, not all tumors or patients respond with equivalent sensitivity. Emerging evidence indicates that targeting Tregs with E7777 to anti-PD-1 as part of combination therapy might change the dynamics of the immune microenvironment, including anti-PD-1 sensitivity, in situations where Tregs are prominent.

Additional human trials will be needed to determine the best administration schedule to maximize host-tumor response with E7777 treatment. Information from the current analysis has already contributed to the study design and dosing regimens of two phase 1 trials, both underway. One study is being conducted in patients with high-risk relapsed/refractory B-cell lymphoma receiving chimeric antigen receptor T-cell (CAR-T) therapy, with the objective of determining the maximally tolerated E7777 dose to use prior to treatment with cyclophosphamide/fludarabine lymphodepletion chemotherapy and CAR-T therapies (NCT04855253). The second is a dose-escalating/dose-finding study to initially evaluate Treg depletion in patients with recurrent or metastatic solid tumors (phase 1A); followed by a cohort of patients with metastatic ovarian cancer (phase 1B) treated with E7777 and the anti-PD-1 agent pembrolizumab (NCT05200559).

In conclusion, this study found that targeting Tregs using E7777 combined with anti-PD-1 (either sequentially or concurrently) demonstrated anti-tumor activity, and transiently and consistently targeted and removed Tregs in the tumor microenvironment. Combination treatment was more effective than monotherapy with either drug and was well tolerated and significantly enhanced long-term survival in solid tumor-bearing animals. The increased Treg cell infiltration induced by anti-PD-1 treatment was counterbalanced by co-treatment with E7777, suggesting potentially synergistic activity. This synergistic activity is being evaluated in human proof-of-concept studies and will inform future clinical trials to assess the safety and efficacy of the combination of E7777 and checkpoint inhibitors in various tumor types.

## Data availability statement

The raw data supporting the conclusions of this article will be made available by the authors, without undue reservation.

## Ethics statement

The animal study was approved by Crown Bioscience Institutional Animal Care and Use Committee. The study was conducted in accordance with the local legislation and institutional requirements.

## Author contributions

HM: Conceptualization, Formal Analysis, Methodology, Writing – review & editing. MW-J: Conceptualization, Data curation, Investigation, Methodology, Supervision, Writing – review & editing. PS: Conceptualization, Formal Analysis, Methodology, Supervision, Writing – original draft, Writing – review & editing. MC: Conceptualization, Formal Analysis, Writing – original draft, Writing – review & editing.
