# Supplementary material for: Targeting regulatory T cells by E7777 enhances CD8 T-cell–mediated anti-tumor activity and extends survival benefit of anti-PD-1 in solid tumor models
Source: Front Immunol. 2023 Oct 27;14:1268979. doi: 10.3389/fimmu.2023.1268979 (PMC10646188; doi:10.3389/fimmu.2023.1268979)
Supplement: Supplementary file 1 [file DataSheet_1.pdf]

Supplementary Material

1 Supplementary Figures and Tables

1.1 Supplementary Figures

Figure S1. Effect of E7777 as single agent or combined with anti-PD-1 on mice body weight change in subcutaneous syngeneic colon cancer model CT-26 (A) and liver cancer model H22 (B). In both models, tumor-bearing mice treated with E7777 had a 10%-15% mean body weight loss during the dosing period, which was recovered during post-dosing observation.

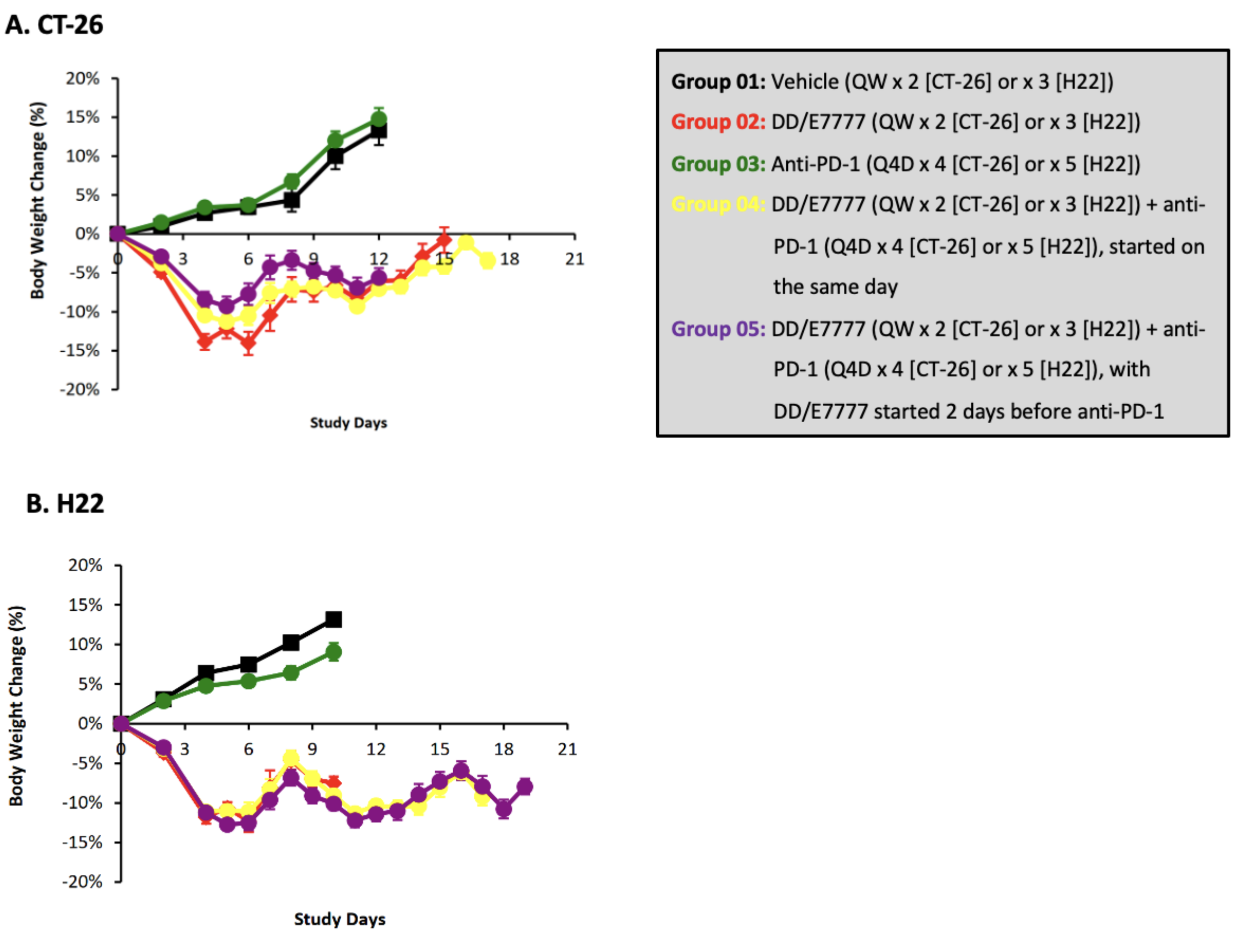

Figure S2. Effect of E7777 on tumor composition (normalized to CD45), evaluating CD3+, CD4+CD8-, CD4-CD8+, and Tregs at study termination for liver cancer model H22, characterized by FC. Immune cell composition was characterized by FC to evaluate changes in multiple T cell subsets resulting from treatment. At study termination, Tregs rebounded in tumors in response to immune stimulation.

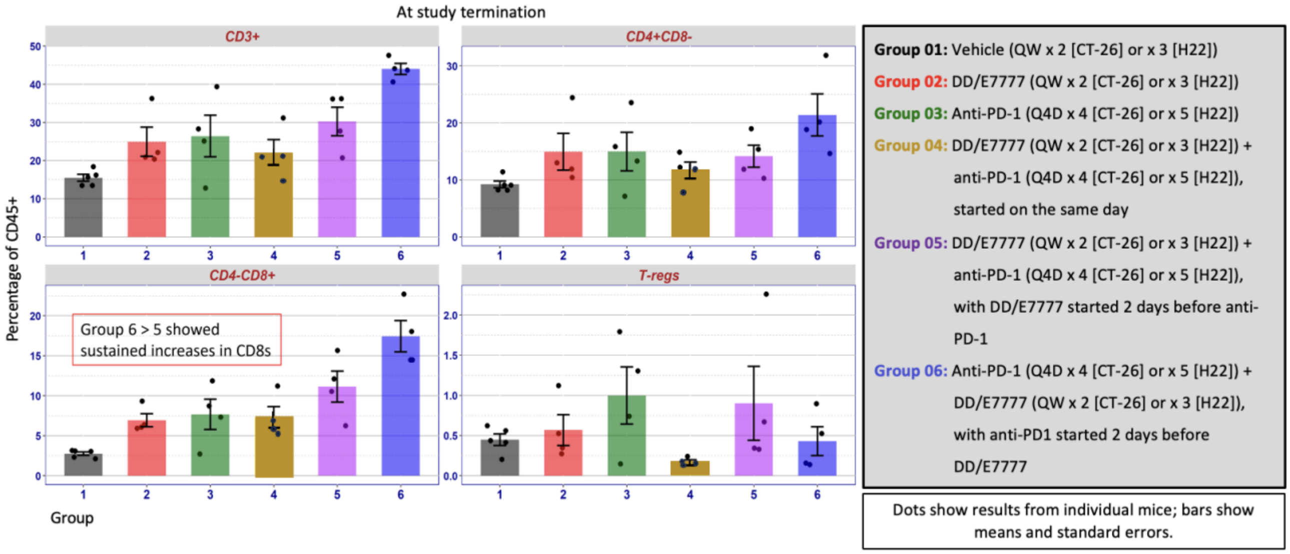

Figure S3. IHC staining of (A) CD8 and (B) FoxP3 within the tumor microenvironment in colon cancer model CT-26, 24 hours after first and second E7777 treatment. Anti-PD-1 treatment increased CD8+ Teff infiltration into tumors, but also induced increased Tregs, which the addition of E7777, on any schedule, helped to inhibit.

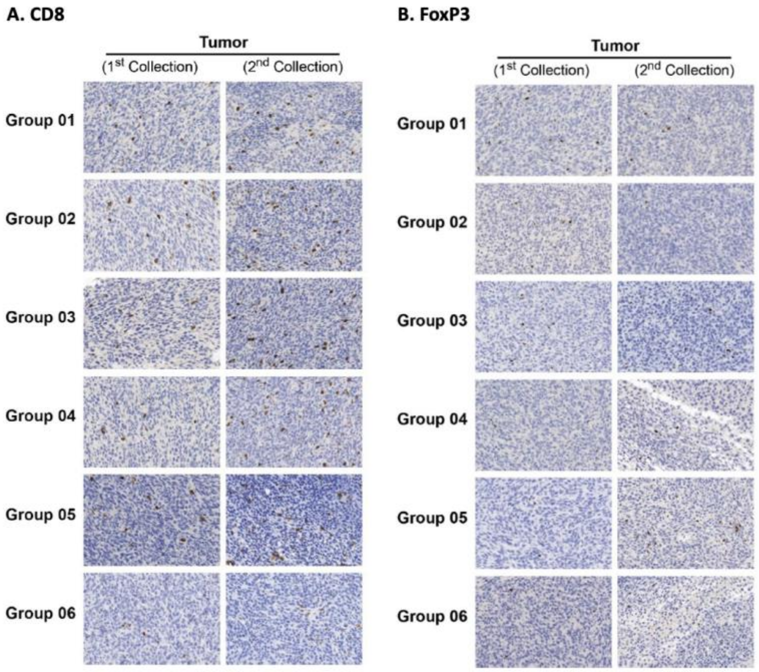

Figure S4. IHC staining of (A) CD8 and (B) FoxP3 within the tumor microenvironment in liver cancer model H22, 24 hours after first and second E7777 treatment. CD8 T-cells in all combination groups increased by more than 2-fold compared to controls by week 2, whereas Tregs decreased by ~2 fold.

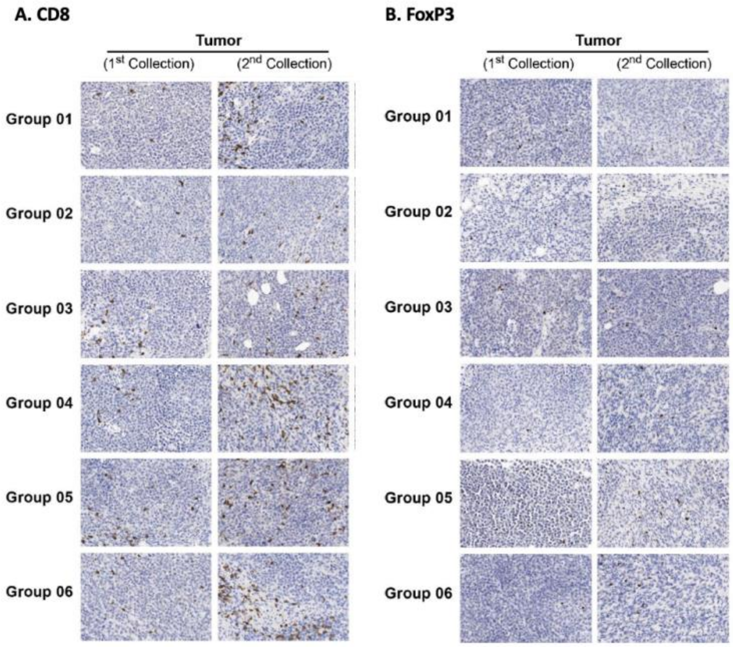

Figure S5. Representative fluorescence activated cell sorting (FACS) plots (A-D) to corroborate flow cytometry-based immune cell characterization shown in Figure 4 and Figure 5.

A. CT-26, Group 3[a]

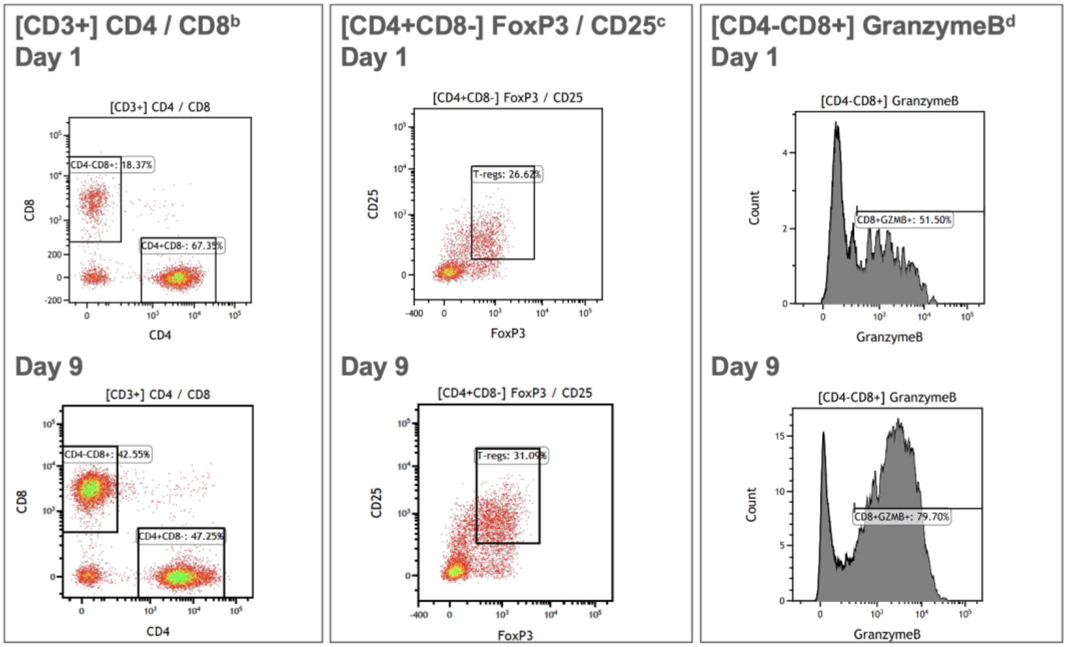

[a] Monotherapy (anti-PD-1 [Q4D x 4]; [b] Represents T helper cells; [c] Represents Treg cells; [d] Represents T active effector cells.

B. CT-26, Group 5[a]

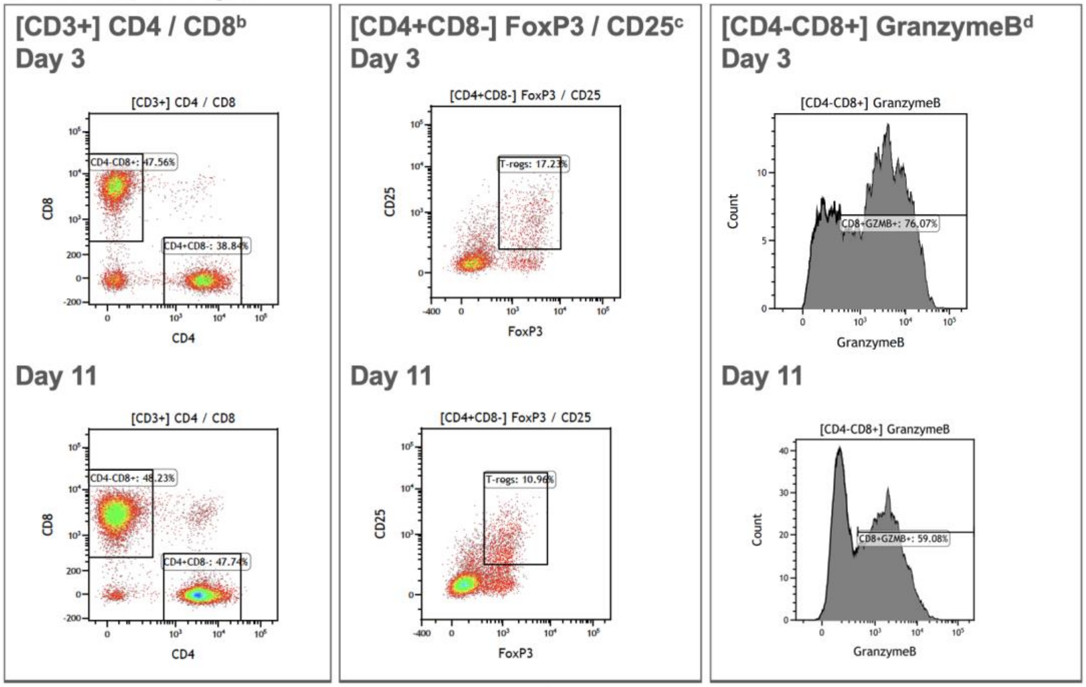

[a] Combination therapy (E7777 [QW x 2] + anti-PD-1 [Q4D x 4]; [b] Represents T helper cells; [c] Represents Treg cells; [d] Represents T active effector cells.

C. H22, Group 3[a]

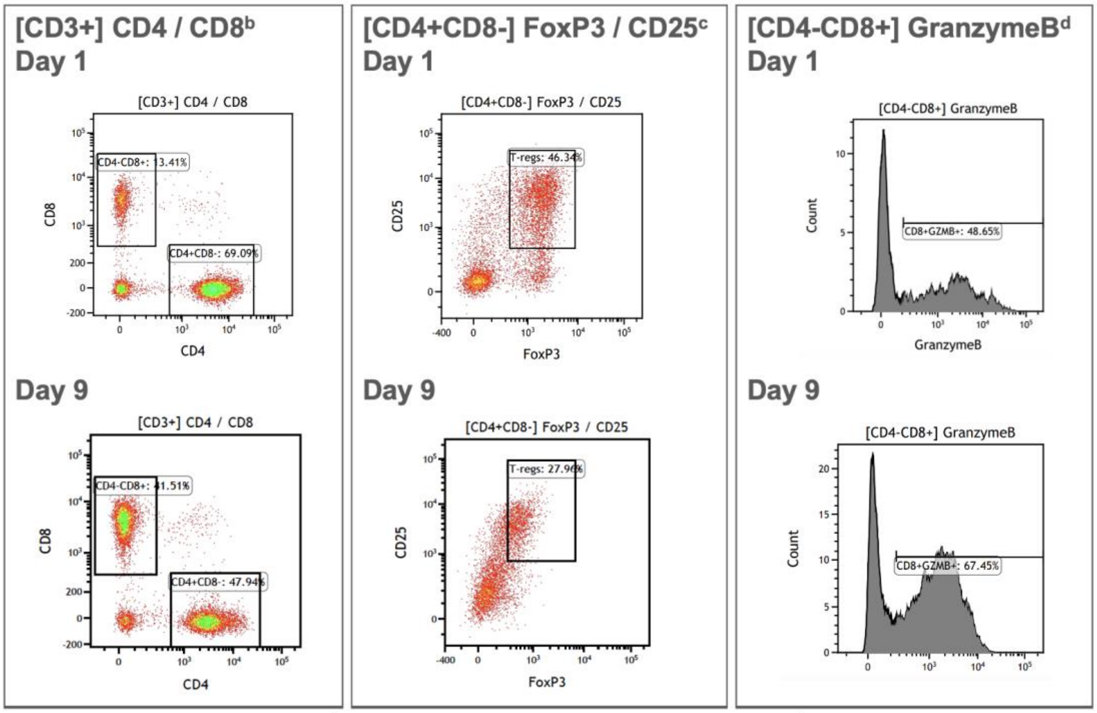

[a] Monotherapy (anti-PD-1 [Q4D x 4]; [b] Represents T helper cells; [c] Represents Treg cells; [d] Represents T active effector cells.

D. H22, Group 5[a]

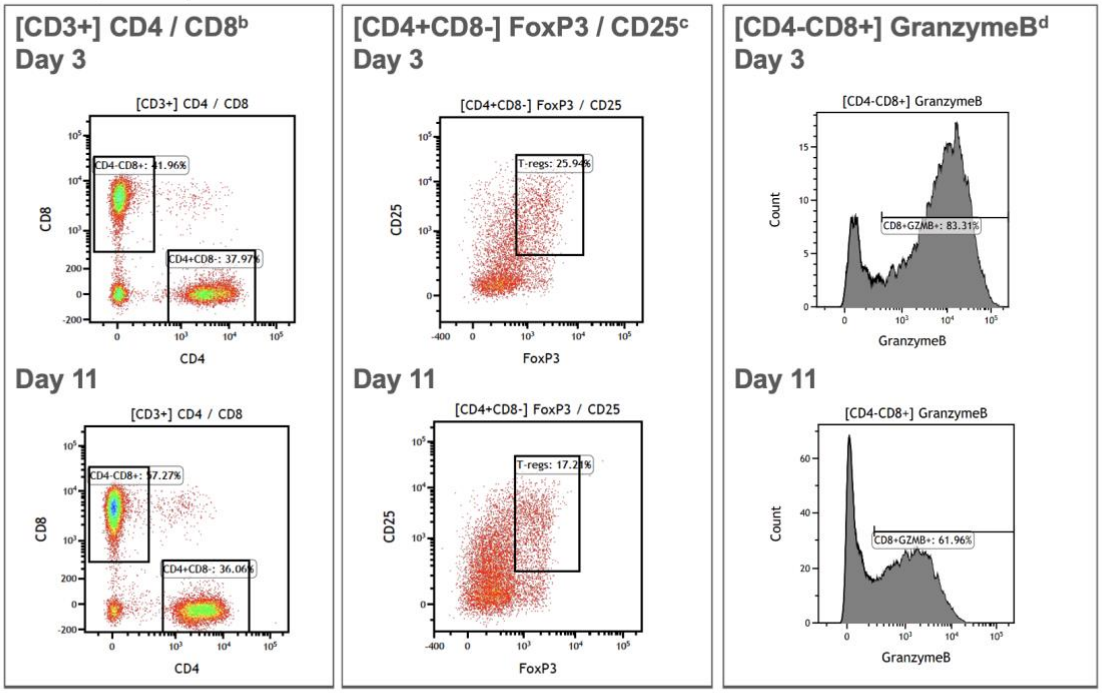

[a] Combination therapy (E7777 [QW x 2] + anti-PD-1 [Q4D x 4]; [b] Represents T helper cells; [c] Represents Treg cells; [d] Represents T active effector cells.

1.2 Supplementary Tables

Table S1. Treatment plans, Experiment 1 and Experiment 2.

Group	N	Treatment	Dose level (µg/mouse)	Dosing solution (mg/mL)	Dosing volume (µL/mouse)	ROA	Dosing frequency & duration	Initial date of dosing
**Experiment 1, CT-26 model study**								
1	24	Vehicle	--	--	100	IV	QW x 2	Day 0
2	24	E7777	2.5	0.025	100	IV	QW x 2	Day 0
3	24	Anti-PD-1	100	1	100	IP	Q4D x 4	Day 0
4	24	E7777	2.5	0.025	100	IV	QW x 2	Day 0
		Anti-PD-1	100	1	100	IP	Q4D x 4	Day 0
5	24	E7777	2.5	0.025	100	IV	QW x 2	Day 0
		Anti-PD-1	100	1	100	IP	Q4D x 4	Day 2
6	24	Anti-PD-1	100	1	100	IP	Q4D x 4	Day 0
		E7777	2.5	0.025	100	IV	QW x 2	Day 2
**Experiment 1, H22 model study**								
1	24	Vehicle	--	--	100	IV	QW x 3	Day 0
2	24	E7777	2.5	0.025	100	IV	QW x 3	Day 0
3	24	Anti-PD-1	100	1	100	IP	Q4D x 5	Day 0
4	24	E7777	2.5	0.025	100	IV	QW x 3	Day 0
		Anti-PD-1	100	1	100	IP	Q4D x 5	Day 0
5	24	E7777	2.5	0.025	100	IV	QW x 3	Day 0
		Anti-PD-1	100	1	100	IP	Q4D x 5	Day 2
6	24	Anti-PD-1	100	1	100	IP	Q4D x 5	Day 0
		E7777	2.5	0.025	100	IV	QW x 3	Day 2
**Experiment 2, CT-26 model study[a]**								
1	16	Vehicle	--	--	100	IV	QW x 3	Day 0
2	16	E7777	2.5	0.025	100	IV	QW x 3	Day 0
3	16	Anti-PD-1	100	1	100	IP	Q4D x 6	Day 0
4	16	E7777	2.5	0.025	100	IV	QW x 3	Day 0
		Anti-PD-1	100	1	100	IP	Q4D x 6	Day 0
5	16	E7777	2.5	0.025	100	IV	QW x 3	Day 0
		Anti-PD-1	100	1	100	IP	Q4D x 6	Day 2
**Experiment 2, H22 model study[a]**								
1	16	Vehicle	--	--	100	IV	QW x 3	Day 0
2	16	E7777	2.5	0.025	100	IV	QW x 3	Day 0
3	16	Anti-PD-1	100	1	100	IP	Q4D x 6	Day 0
4	16	E7777	2.5	0.025	100	IV	QW x 3	Day 0
		Anti-PD-1	100	1	100	IP	Q4D x 6	Day 0
5	16	E7777	2.5	0.025	100	IV	QW x 3	Day 0
		Anti-PD-1	100	1	100	IP	Q4D x 6	Day 2

a. E7777 and vehicle were dosed on Day 0, 8 and 15.
IP, intraperitoneal; IV, intravenous; ROA, route of administration; Q4D, every 4 days (1 day dosing and 3 days off); QW, once a week.

Table S2. Survival analysis of E7777 as single agent or combined with anti-PD-1 in subcutaneous syngeneic liver cancer model H22 and colon cancer model CT26.

Group	Treatment	MST (days)	ILS (%)	*P*- value[a]
**CT-26 model study**				
1	Vehicle, IV, QW x 3	15	-	-
2	E7777, 2.5 µg/mouse, IV, QW x 3	22	46.7	<0.05
3	Anti-PD-1, 100 µg/mouse, IP, Q4D x6	19.5	26.7	<0.05
4	E7777, 2.5 µg/mouse, IV, QW x 3<br>Anti-PD-1, 100 µg/mouse, IP, Q4D x6	38	153.3	<0.001
5	E7777, 2.5 µg/mouse, IV, QW x 3<br>Anti-PD-1, 100 µg/mouse, IP, Q4D x6 from Day 2	52	246.7	<0.001
**H22 model study**				
1	Vehicle, IV, QW x 3	12	-	-
2	E7777, 2.5 µg/mouse, IV, QW x 3	23	91.7	<0.001
3	Anti-PD-1, 100 µg/mouse, IP, Q4D x6	16	33.3	<0.05
4	E7777, 2.5 µg/mouse, IV, QW x 3<br>Anti-PD-1, 100 µg/mouse, IP, Q4D x6	85.5	612.5	<0.001
5	E7777, 2.5 µg/mouse, IV, QW x 3<br>Anti-PD-1, 100 µg/mouse, IP, Q4D x6 from Day 2	80.5	570.8	<0.001

a. Compared with vehicle control group using log-rank test.
IP, intraperitoneal; ILS, increase in life span; IV, intravenous; MST, median survival time; Q4D, every 4 days (1 day dosing and 3 days off); QW, once a week.

Table S3. Antitumor activity of E7777 as single agent or combined with anti-PD-1 in subcutaneous syngeneic liver cancer model H22 and colon cancer model CT-26, Experiment 2.

Group	Treatment	Tumor Size (mm^3)[a] on Day 12	T/C (%) on Day 12	TGI (%) on Day 12	*P*-value[b]	CR ratio
**CT-26 model study**						
1	Vehicle, IV, QW x 3	2039.3±234.7	-	-	-	0/16
2	E7777, 2.5 µg/mouse, IV, QW x 3	1612.5±157.7	79.1	20.9	>0.05	0/16
3	Anti-PD-1, 100 µg/mouse, IP, Q4D x6	1844.3±236.3	90.4	9.6	>0.05	0/16
4	E7777, 2.5 µg/mouse, IV, QW x 3<br>Anti-PD-1, 100 µg/mouse, IP, Q4D x6	689.7±109.8	33.8	66.2	<0.001	5/16
5	E7777, 2.5 µg/mouse, IV, QW x 3<br>Anti-PD-1, 100 µg/mouse, IP, Q4D x6 from Day 2	940.1±169.7	46.1	53.9	<0.001	5/16
**H22 model study**						
1	Vehicle, IV, QW x 3	3029.4±249.9	-	-	-	0/16
2	E7777, 2.5 µg/mouse, IV, QW x 3	1640.3±143.3	54.1	45.9	<0.01	0/16
3	Anti-PD-1, 100 µg/mouse, IP, Q4D x6	1875.2±280.6	61.9	38.1	<0.01	0/16
4	E7777, 2.5 µg/mouse, IV, QW x 3<br>Anti-PD-1, 100 µg/mouse, IP, Q4D x6	1216.6±125.0	40.2	59.8	<0.001	8/16
5	E7777, 2.5 µg/mouse, IV, QW x 3<br>Anti-PD-1, 100 µg/mouse, IP, Q4D x6 from Day 2	896.6±76.8	29.6	70.4	<0.001	8/16

a. Mean ± standard error of the mean.
b. Compared with Group 1 tumor volume on Day 12 using Conover's non-parametric test.
CR, tumor complete response (tumor regressed to 0); IP, intraperitoneal; IV, intravenous; Q4D, every 4 days (1 day dosing and 3 days off); QW, once a week; T/C, ratio of tumor volume in treated versus control mice; TGI, tumor growth inhibition.
